# Hemodynamic Transesophageal Echocardiography-Guided Venous-Arterial Extracorporeal Membrane Oxygenation Support in a Case of Giant Cell Myocarditis

**DOI:** 10.1155/2016/5407597

**Published:** 2016-08-25

**Authors:** Juan G. Ripoll, Robert A. Ratzlaff, David M. Menke, Maria C. Olave, Joseph J. Maleszewski, José L. Díaz-Gómez

**Affiliations:** ^1^Department of Critical Care Medicine, Mayo Clinic, 4500 San Pablo Road, Jacksonville, FL, USA; ^2^Department of Anesthesiology, Mayo Clinic, 4500 San Pablo Road, Jacksonville, FL, USA; ^3^Department of Pathology, Mayo Clinic, 4500 San Pablo Road, Jacksonville, FL, USA; ^4^Division of Anatomic Pathology, Mayo Clinic, 200 First St SW, Rochester, MN, USA; ^5^Department of Neurosurgery, Mayo Clinic, 4500 San Pablo Road, Jacksonville, FL, USA

## Abstract

Giant cell myocarditis (GCM) is a rare and commonly fatal form of fulminant myocarditis. During the acute phase, while immunosuppressive therapy is initiated, venoarterial extracorporeal membrane oxygenation (VA-ECMO) support is commonly used as a bridge to heart transplantation or recovery. Until recently, conventional transesophageal echocardiography and transthoracic echocardiography were the tools available for hemodynamic assessment of patients on this form of mechanical circulatory support. Nevertheless, both techniques have their limitations. We present a case of a 54-year-old man diagnosed with GCM requiring VA-ECMO support that was monitored under a novel miniaturized transesophageal echocardiography (hTEE) probe recently approved for 72 hours of continuous hemodynamic monitoring. Our case highlights the value of this novel, flexible, and disposable device for hemodynamic monitoring, accurate therapy guidance, and potential VA-ECMO weaning process of patients with this form of severe myocarditis.

## 1. Introduction

Giant cell myocarditis (GCM) is a rare clinical condition characterized by rapid compromise of cardiac systolic function, ultimately leading to severe cardiogenic shock. It has a grave prognosis with a rate of death or heart transplantation of 70% at 1 year. Recently, venoarterial extracorporeal membrane oxygenation (VA-ECMO) has been used as a bridge to cardiac transplantation or recovery [1, 2]. Although no current guidelines are available for an optimal monitoring device for patients under extracorporeal membrane oxygenation (ECMO) support, conventional transesophageal echocardiography (TEE) or transthoracic echocardiography (TTE) is commonly used for this purpose [3]. Nevertheless, both techniques have limitations [4, 5]. We present a case of fulminant GCM under VA-ECMO support monitored with a novel, miniaturized, flexible, and disposable hemodynamic transesophageal echocardiography (hTEE) probe that allows for 72 hours of continuous hemodynamic monitoring.

## 2. Case Presentation

A 54-year-old man with a history of psoriatic arthritis, migraines, osteoarthritis, and hyperlipidemia presented to a primary care facility with complaints of sudden generalized weakness and dizziness. The initial assessment was remarkable for elevated serial troponins and ST elevation in the inferior echocardiogram leads (V2, V3, and aVF). He was transferred to a tertiary care hospital for further management of his cardiac condition.

Upon arrival, he underwent a cardiac catheterization that revealed clear coronary arteries. A subsequent echocardiography displayed a left ventricular ejection fraction of 30%. Despite proper management, the patient experienced a third-degree atrioventricular block requiring the implantation of a dual chamber pacemaker without defibrillator capabilities. After full hemodynamic recovering, the patient was discharged and returned to his daily activities.

Three days later, he was readmitted to the same tertiary care hospital after experiencing 2 syncopal episodes, chest discomfort, and blurry vision. Further clinical studies demonstrated no additional cardiac abnormalities, and a computed tomography scan with angiography of the head, neck, and chest was unremarkable. Autoimmune and infectious diseases tests (including Lyme disease) and a lumbar puncture test were also negative.

The night he was discharged, the patient experienced progressively worsening dyspnea and another syncopal episode. He was readmitted tachycardic (heart rate > 120 bpm), normotensive (blood pressure 110/60 mmHg), tachypneic (respiratory rate > 20 rpm), and diaphoretic, with elevated troponin I levels (10.7 ng/mL) and a positive D-dimer. A second cardiac catheterization was performed in addition to an extensive diagnostic workup for pulmonary embolism. Both diagnostic tests were negative, and the patient's hemodynamics started to deteriorate. He was initiated on vasopressor therapy (dobutamine) but developed rapid ventricular tachycardia requiring antiarrhythmic medication (amiodarone). Once the cardiac rhythm was controlled, he underwent an intra-aortic balloon pump insertion and was transferred to our institution for possible ECMO support.

The initial evaluation was notable for mixed cardiogenic and vasodilatory shock with associated acute kidney injury, metabolic acidosis, acute liver failure, coagulopathy, and acute anemia (Table 1). TTE revealed severe left ventricular systolic dysfunction with an estimated left ventricular ejection fraction of 25% and a concomitant severe right ventricular dysfunction. Due to the high clinical suspicion of GCM, an attempt of endomyocardial biopsy (EMB) was performed. However, the procedure was complicated by rapid ventricular tachycardia and inability to obtain endomyocardial samples.

As a result of incessant slow ventricular tachycardia with spikes of rapid ventricular tachycardia, an elective intubation with direct current cardioversion at 200 J was initiated. Following the procedure, stabilization of mean arterial pressure was achieved. High-dose steroids and antithymocyte-globulin were empirically initiated for a likely diagnosis of GCM. No initial immunosuppressive therapy was considered because of the patient's severe multiorgan compromise.

The day after admission, the intra-aortic balloon pump was removed and VA-ECMO (via left femoral artery-left femoral vein) was initiated as a bridge to cardiac transplantation. A successful intraoperative EMB confirmed the diagnosis of GCM.

As the patient's kidney function continued to deteriorate, he was started on continuous venous-venous hemodialysis. Therefore, the selected immunosuppressive therapy was mycophenolate rather than tacrolimus.

After immunosuppressive therapy was started, the patient developed fever and purulent secretions. Cultures from a bronchoalveolar lavage revealed the presence of Gram-negative bacilli (*Escherichia coli*). Septic shock, likely a result of pneumonia, was considered, and wide-spectrum antibiotics were initiated.

**Figure 1:**
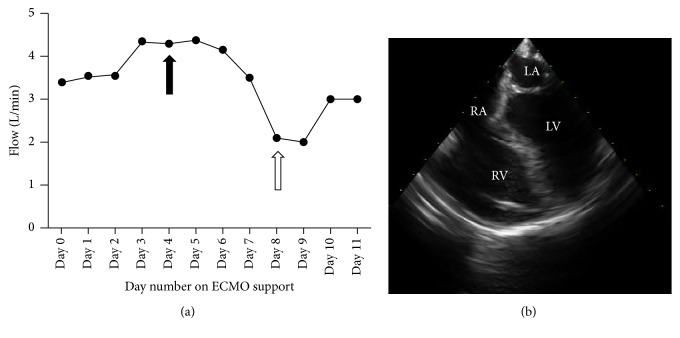
hTEE monitoring of VA-ECMO support. hTEE assessments were performed on day 4 (black arrow) and day 8 (white arrow), respectively (a). A mid-esophageal four-chamber view (day 11) revealed persistent biventricular systolic dysfunction despite VA-ECMO support (b).

In the setting of this multifactorial shock (cardiogenic, septic), the hemodynamic status of the patient continued to deteriorate. To better characterize the patient's state of shock and to guide inotropic, vasopressor, and fluid therapy, an initial 72-hour continuous hTEE evaluation was performed. Persistent, severe, right ventricular, and moderate left ventricular dysfunctions were shown. Transfusions of blood products and vasopressor therapy adjustment were decided. As tolerated by the patient, hTEE-guided weaning from VA-ECMO was considered (Figure 1(a)).

Four days later, a second hTEE examination was performed (Figure 1(a)) in order for the cardiology, cardiothoracic surgery, and critical care teams to reassess the patient's heart function and make a decision about weaning the patient from VA-ECMO support. Unfortunately, no signs of cardiac function recovery were identified with hTEE after 11 days of VA-ECMO support (Figure 1(b)). Consequently, the patient was unable to tolerate the definitive weaning trial.

**Figure 2:**
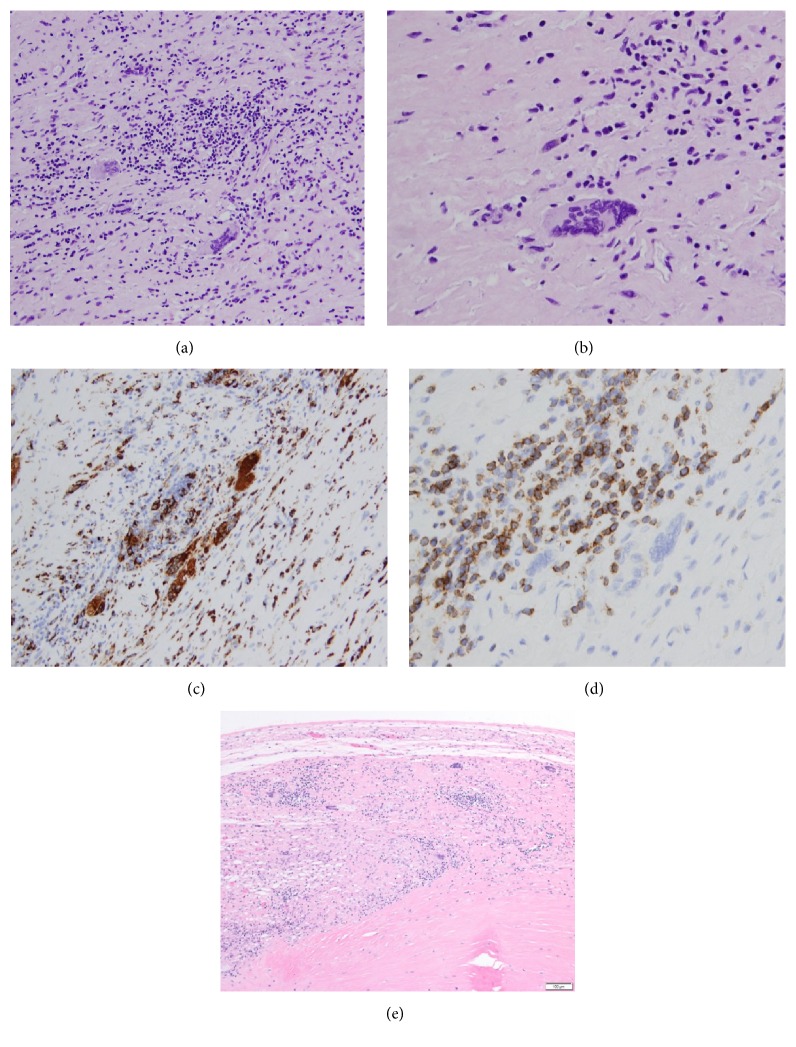
Histopathology of giant cell myocarditis. (a) Myocardium with prominent lymphohistiocytic infiltrate and well-formed multinucleated giant cells (H&E, original magnification ×200). (b) High power magnification (H&E, original magnification ×400) showing extensive myocardial damage by a dense inflammatory infiltrate. (c) Histiocytic infiltrate (CD68-PGM-1, original magnification ×200). (d) The lymphocytic infiltrate consists primarily of T-lymphocytes (CD3, original magnification ×400). (e) Atrioventricular node involved by giant cell myocarditis (H&E, original magnification, ×100).

Due to his underlying multisystem organ failure, the patient was not deemed a candidate for heart transplantation or for placement of a left ventricular assist device or a biventricular assist device. Thus, the patient's family was consulted, and compassionate withdrawals of all measures were initiated. Under family consent, a chest autopsy further confirmed the diagnosis of GCM (Figure 2).

## 3. Discussion

Idiopathic GCM is a rare and fatal form of T-cell mediated inflammatory myocarditis with an estimated incidence between 6.6 and 23.4 cases per 100.000 individuals [6]. It predominantly affects young people with slight male preponderance. Up to 8% of affected patients have concomitant inflammatory bowel disease (ulcerative colitis or Crohn disease) [7]. The most common clinical manifestations of GCM include rapidly progressive heart failure (75%) and incessant ventricular arrhythmias (14%). A syndrome mimicking acute myocardial infarction (6%) and complete heart block (5%) are among the uncommon clinical presentations of the disease. Diagnosis of GCM relies on EMB showing a diffuse multifocal inflammatory infiltrates with associated myocardial necrosis, presence of multinucleated giant cells, and an absence of sarcoid-like granulomas [7, 8].

Immunosuppressive therapy is a well-established treatment for GCM [9]. On contemporary regimens, two-thirds of patients reached a partial clinical remission characterized by transplant-free survival and reduced risk of severe heart failure [10]. However, there is no data available regarding the maintenance of remission under long-term immunosuppressive therapy. Thus, heart transplantation still remains the definitive treatment for GCM [7, 11].

Acute heart failure is the most common clinical manifestation of GCM. Immunosuppressive agents need time to be effective; meanwhile, cardiovascular support must be assured. Thus, mechanical circulatory devices are valuable alternatives as a bridge both to cardiac transplantation and to myocardial recovery [12–14]. Currently, VA-ECMO is considered a well-known bridging therapy in the setting of fulminant GCM [2, 15]. Although no guidelines are currently available, ECMO monitoring has been commonly performed under TTE or TEE guidance [3]. Nevertheless, several limitations arise with the use of these technologies. On one hand, in the intensive care unit setting, TTE diagnostic performance is considered inferior to TEE due to poorly discernible echocardiographic windows in mechanically ventilated patients [4]. On the other hand, TEE requires highly trained clinicians (cardiac anesthesiologists or cardiologists), the examination is discontinuous in nature, and the need for multiple probe insertions could potentially lead to major injuries such as esophageal trauma and bleeding [4, 5].

To overcome these limitations, a flexible, disposable, and miniaturized hTEE probe has been approved by the Food and Drug Administration. The device can be utilized continuously for up to 72 hours and provides a real-time qualitative and semiquantitative assessment of sudden hemodynamic changes [16]. Simplified insertion and improved tolerance are among the potential benefits for hemodynamically unstable patients requiring mechanical ventilation [17, 18]. In the intensive care unit setting, hTEE provides supplementary information to invasive monitoring [19] and displays good interrater reliability when performed by nonexperienced operators [20]. Thus, hTEE theoretically provides a safer, faster, and more user-friendly assessment of hemodynamic status compared to TTE and continuous TEE.

Only 1 previous study of 21 patients with underlying cardiogenic shock demonstrated the use of hTEE as a monitoring tool for ECMO weaning [21]. To the best of our knowledge, this is the first case reporting the use of an hTEE-guided approach to assess a severe cardiogenic shock in a case of fulminant GCM.

Although our patient ultimately expired as a result of severe multiorgan failure, there are multiple reasons to routinely implement hTEE examination as a monitoring tool in critically ill patients requiring VA-ECMO support. First, this imaging modality allows for 72 hours of continuous monitoring, leading to optimal management of fluid therapy and vasopressor titration. Second, it allows for prompt recognition of sudden cardiac complications emerging from the progressive cardiac damage displayed in disease states, such as GCM. Finally, hTEE provides a real-time assessment of cardiac structures, permitting a rapid screening of signs of cardiac recovery in patients under VA-ECMO support and thus favoring the weaning process [21].

In our case, close monitoring of VA-ECMO support with hTEE allowed us to better characterize a complex state of shock (cardiogenic and vasodilatory) [19]. Hence, we have a more appropriate initial resuscitation in the early phase of our patient's care. Furthermore, we present the usefulness of hTEE for ECMO weaning trial. Although in this case it was utilized for decision-making of withdrawal of life support, it would potentially guide a final decision of ECMO explantation after treatment of severe refractory cardiogenic and septic shock. Although this hTEE management did not lead to better outcome, it can be considered as a valuable tool to a more prompt characterization of complex state of shock since presentation and guiding trials of ECMO support weaning in entities with a high lethality rates like GCM.

## 4. Conclusion

Our case highlights that an hTEE-guided approach is a valuable alternative for the hemodynamic assessment of patients with GCM under VA-ECMO support. Increased monitoring of mechanical circulatory support in complex states of shock could potentially lead to more accurate clinical decisions, including decision for therapy management, ECMO weaning, and even timely withdrawal of all life-support measures in severely compromised individuals.

## Figures and Tables

**Table 1:** Overview of notable admission laboratory data.

Admission laboratory data	
*General chemistry*	
Sodium (Na), mEq/L	131
Potassium (K), mEq/L	4.8
Creatinine (mg/dL)	2.6
Lactate (mmol/L)	4.9
Aspartate aminotransferase (AST) (units per liter)	6693
Alanine aminotransferase (ALT) (units per liter)	4040
B-type natriuretic peptide (BNP) (pg/mL)	960

*Blood cell count and differential*	
Hemoglobin (g/dL)	10.8
Hematocrit (%)	32.6
Neutrophils (absolute number/% neutrophils)	18.760/92.1

*Blood gases*	
PH arterial	7.425
PaCO_2_ (mmHg)	22.9
Bicarbonate (mEq/L)	14.7
SaO_2_ (%)	97.4
SvO_2_ (%)	55.8

*Coagulation studies*	
aPTT (sec)	42.3
INR	2.0
Prothrombin time (sec)	23

## Abbreviations

ECMO: Extracorporeal membrane oxygenation
EMB: Endomyocardial biopsy
GCM: Giant cell myocarditis
hTEE: Hemodynamic transesophageal echocardiography
TEE: Transesophageal echocardiography
TTE: Transthoracic echocardiography
VA-ECMO: Venoarterial extracorporeal membrane oxygenation.
